# Pancreatic Ductal Adenocarcinoma: A Strong Imbalance of Good and Bad Immunological Cops in the Tumor Microenvironment

**DOI:** 10.3389/fimmu.2018.01044

**Published:** 2018-05-14

**Authors:** Etienne D. Foucher, Clément Ghigo, Salem Chouaib, Jérôme Galon, Juan Iovanna, Daniel Olive

**Affiliations:** ^1^Team Immunity and Cancer, CRCM, Aix Marseille Univ, CNRS, INSERM, Institut Paoli-Calmettes, Marseille, France; ^2^Team Cellular Stress, CRCM, Aix Marseille Univ, CNRS, INSERM, Institut Paoli-Calmettes, Marseille, France; ^3^INSERM UMR1186, Integrative Tumor Immunology and Genetic Oncology, Gustave Roussy, Equipe Labellisée par La Ligue Contre Le Cancer, EPHE, Faculté de Médecine, Université Paris-Sud, Université Paris-Saclay, Villejuif, France; ^4^Thumbay Research Institute for Precision Medicine, Gulf Medical University, Ajman, United Arab Emirates; ^5^Laboratory of Integrative Cancer Immunology, INSERM, UMRS1138, Paris, France

**Keywords:** pancreatic ductal adenocarcinoma, immune infiltrate, tumor microenvironment, immunosuppression, hypoxia, immune checkpoint

## Abstract

Pancreatic ductal adenocarcinoma (PDAC) is one of the most aggressive and lethal cancers with very few available treatments. For many decades, gemcitabine was the only treatment for patients with PDAC. A recent attempt to improve patient survival by combining this chemotherapy with FOLFIRINOX and nab-paclitaxel failed and instead resulted in increased toxicity. Novel therapies are urgently required to improve PDAC patient survival. New treatments in other cancers such as melanoma, non-small-cell lung cancer, and renal cancer have emerged, based on immunotherapy targeting the immune checkpoints cytotoxic T-lymphocyte-associated antigen 4 or programmed death 1 ligand. However, the first clinical trials using such immune checkpoint inhibitors in PDAC have had limited success. Resistance to immunotherapy in PDAC remains unclear but could be due to tissue components (cancer-associated fibroblasts, desmoplasia, hypoxia) and to the imbalance between immunosuppressive and effector immune populations in the tumor microenvironment. In this review, we analyzed the presence of “good and bad immunological cops” in PDAC and discussed the significance of changes in their balance.

## Introduction

Pancreatic ductal adenocarcinoma (PDAC) is the fourth-leading cause of cancer-related death in the world, with a 5-year survival rate of less than 5%. Each year more than 350,000 people worldwide are diagnosed and more than 340,000 die of the disease. The incidence is rising, and some reports project an over twofold increase in the number of new PDAC cases and PDAC deaths by 2030 (1).

The only curative treatment is complete surgical resection. Unfortunately, fewer than 20% of patients are candidates for surgery since their cancer has usually already spread before diagnosis. For this small subgroup of patients undergoing surgery, adjuvant treatment with the chemotherapy drug gemcitabine, Erlotinib, or more recently FOLFIRINOX has been shown to slightly improve survival (2, 3).

It appears that tumors develop multiple immunosuppressive mechanisms to down-regulate the innate and effector arms of the immune system, thus compromising most of the immunotherapeutic strategies that have been proposed during the last decade. In PDAC, the tumor microenvironment (TME) seems to play a pivotal role in tumor escape. A large number of cells or mechanisms participate together to improve the proliferation of tumor cells (4, 5). One of these is immune cells themselves, in particular immunosuppressive leukocytes that we will discuss in this review. Other components contribute toward PDAC cancerogenesis such as cancer-associated fibroblasts (CAFs) and extracellular matrix proteins. Together, these components interact with tumor cells to develop a pro-tumor environment and support proliferation. Another important mechanism called hypoxia exerts a strong impact on the structure of the tumor tissue (angiogenesis) and also on cells in the TME where hypoxia induces the development of immunosuppressive cell populations. Together these components participate toward inducing the desmoplastic reaction in the TME, which increases the “sealing off” (high level of intra-tumor blood vessel pressure) from effector immune cells (failing upon immune cell recruitment) and from drug delivery (chemoresistance) (6).

In this review, we focus on the organization and the role of infiltrating anti- or pro-tumor immune cell populations (referred to as “good and bad immunological cops,” respectively) during the course of PDAC and discuss the state-of-the-art of immunotherapy in PDAC.

## Inflammation and Immune Cell Infiltrate in the TME

**Figure 1:**
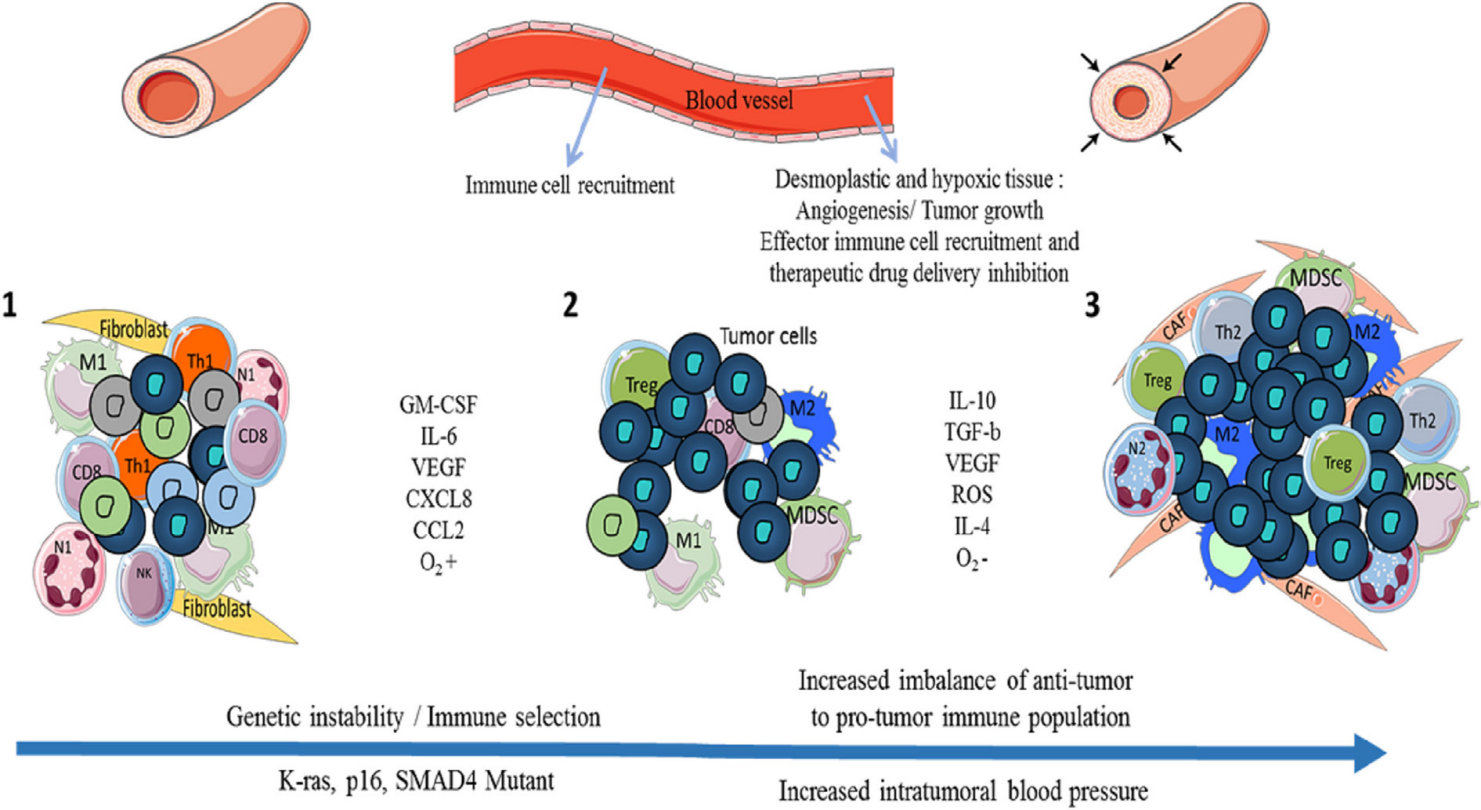
Evolution of the immune cell population and pancreatic ductal adenocarcinoma (PDAC) development through the three Es of cancer immunoediting. During cancer immunosurveillance (1), immune effector cells M1 macrophages and N1 neutrophils are recruited to the tissue in order to eliminate heterogenic mutant/tumor cells. While these immune cells kill most tumor cells, specific resistant tumor clones (in dark blue) survive (2). An equilibrium between anti- and pro-tumor immune cells is maintained until tumor cells and immunosuppressive immune cells develop tumor escape mechanisms via the secretion of pro-tumor factors (IL-10, TGF-β, etc.) and inhibitory co-signaling molecules (3). Tumor escape induces the growth of tumor cells, angiogenesis, metastasis, the establishment of an immunosuppressive microenvironment with the presence of Tregs, tumor-associated macrophages (TAMs) such as M2, CAFs, myeloid-derived suppressive cells (MDSCs), tumor-associated neutrophils (TANs) such as N2 and with hypoxia and desmoplasia, which increase the pro-tumor impact and create a barrier (high blood pressure) against therapeutic drug delivery and recruitment of effector immune cells. M1: anti-tumor macrophages, M2: pro-tumor macrophages, N1: anti-tumor neutrophils, N2: pro-tumor neutrophils, CD8: CD8^+^ T cells, Th1/Th2: CD4^+^ Th1 (anti-tumor) or Th2 (pro-tumor) T cells, Treg: regulatory T cells, CAFs: cancer-associated fibroblasts.

**Figure 2:**
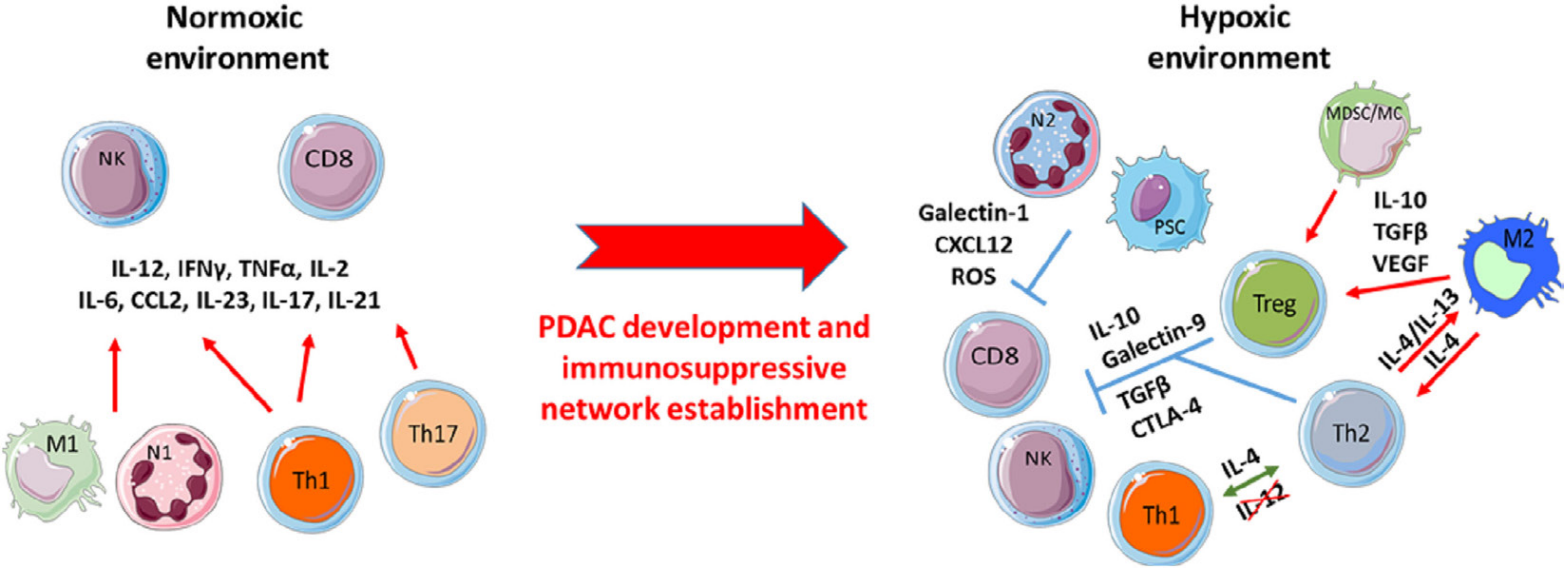
Pancreatic ductal adenocarcinoma (PDAC) development induces the shift of pro-inflammatory to immunosuppressive immune populations. At early PDAC stages, a primarily anti-tumor immune population favors the effector T-cell functions of such as CD8^+^ and natural killer (NK) cells toward the prevention of tumor cell growth. Despite this initial anti-tumor response, over time chronic activation of these effector immune cells brings about a smoldering inflammation process that selects resistant tumor clones resulting in the promotion and development of immunosuppressive immune populations under hypoxic stress.

The link between tumor growth and inflammation has been greatly illustrated in the literature. The three Es (Elimination/Equilibrium/Escape) of cancer immunoediting perfectly reflect the development of pancreatic cancer and the immune population evolution in the TME (7). While inflammation is classically associated with an anti-tumor Th1 immune response (cancer immunosurveillance/elimination phase), tumor-associated inflammation is chronic, smoldering, and detrimental and participates toward tumor cell development and the accumulation of immunosuppressive leukocytes (equilibrium and escape phases) (Figure 1). In some cancers such as PDAC, Kras or myc oncogenes are responsible for such chronic and smoldering inflammation in the TME (8, 9). Regardless of origin, this inflammation allows cancer cells to establish the tumor escape and development processes (10, 11). In PDAC, despite the hypoxia and hyaluronan-induced development of desmoplastic stroma, the TME is composed of several immune cell populations (12). At early stages, effector cells such as natural killer (NK) cells, CD8^+^ T cells, and CD4^+^ T cells can be present and activated. Nevertheless, during the selection of resistant tumor cells (during the elimination process) and the development of the escape mechanism, the TME induces the recruitment of monocytes and neutrophils, which then have acquired an anti-inflammatory phenotype (M2 and N2 respectively), the recruitment of myeloid-derived suppressive cells (MDSCs), the recruitment and/or the polarization of regulatory T cells (Tregs) or Th17, and the recruitment of Th1 to Th2 cell shift (13, 14). Furthermore, CD8^+^ T cells, NK cells, and dendritic cells are deactivated or exhausted in order to inhibit anti-tumor function. Of course, the transformation of pro-inflammatory to anti-inflammatory in the TME increases the tumor growth and angiogenesis and correlates with poor survival (Figures 1 and 2) (13).

## Good Cops

### Effector Immune Cells

#### CD8^+^ T Cells

Tumor infiltrated CD8^+^ T cells (also called cytotoxic T lymphocytes; CTLs) are immune effector cells that can kill cancer cells using perforin and granzyme molecules. Analysis in peripheral blood has revealed significantly decreased circulating CTLs and lower perforin expression levels in pancreatic cancer patients compared with healthy controls (15). Immunohistochemistry on pancreatic cancer samples showed a higher cellular infiltration compared to normal pancreas and survival studies have shown that higher levels of tumor infiltrating CD4^+^ and CD8^+^ T cells are associated with longer survival (16).

Shortly after T-cell activation, cytotoxic T-lymphocyte-associated antigen 4 (CTLA4) is translocated to the plasma membrane. This co-receptor molecule binds to B7 ligand with a higher affinity than does the co-receptor CD28, leading to inhibition of the T-cell activation. Furthermore, PDAC cells express PD-L1, which binds to PD1 expressed on activated T cells (17). Interaction between these molecules leads to T-cell anergy or death and consequently promotes tumor progression (18).

The restoration of exhausted CD8^+^ T cells and recovery of their effector role represent one of the main therapeutic objectives toward the destruction of cancer cells.

#### CD4^+^ T Cells

CD4^+^ T cells (T helper cells) play an important role in the immune response by secreting several cytokines that modulate the function of B and CD8^+^ T cells. Their peripheral blood levels are reduced in patients with pancreatic cancer compared to healthy controls (19). Naive CD4^+^ T cells can differentiate into the following two main subsets: Th1 cells, which support cell-mediated immune responses by secreting IL-2 and IFN-γ (activate macrophages and CD8^+^ T-cell proliferation), and Th2 cells, which induce humoral immune responses by secreting IL-4, IL-5, IL-6, IL-9, IL-10, and IL-13 (stimulate B-cell proliferation and induce B-cell antibody class switching) (20). In PDAC cancer, the shift from Th1 to Th2 cells is correlated to reduced survival.

#### NK Cells

Natural killer cells are cytotoxic lymphocytes of the innate immune system. Activation of these cells is determined by the balance between activating and inhibitory receptor stimulation. Analysis of peripheral blood mononuclear cells has revealed reduced levels of NK cells in patients with PDAC compared to healthy controls (19). Patients were also found to have significantly lower levels of two activating receptors (CD226 and CD96) on their circulating NK cells compared to healthy controls (21). The decrease in the level of activating receptors on NK cells could indicate dysfunction of these cells and may represent a factor promoting PDAC progression. These data suggest that reactivation of NK cells via these activator receptors could be a new target for cancer immunotherapy.

## Bad Cops

### Anti-Inflammatory Myeloid Cells

#### Tumor-Associated Macrophages (TAMs)

Monocytes recruited to the tumor site can differentiate into TAMs. In the majority of solid tumors such as in PDAC, TAMs represent the most abundant immune population in the TME. Tumor cells express many factors including CCL2 (under hypoxic conditions), M-CSF or GM-CSF, IL-10, TGF-β, and IL-6, all of which favor the recruitment and generation of TAMs (22). At early cancer stages, TAMs can be polarized into an anti- (M1) or pro- (M2) tumor phenotype (23, 24), whereas at advanced stages, they are mainly present as the M2 subtype (CD14^+^ CD163^+^) (23) and their presence is associated with bad prognosis in PDAC (25, 26). As reported by Cui et al, TAMs play large roles in the promotion of tumor growth and development of an immunosuppressive microenvironment. They do this by secreting angiogenic factors (IL-6, VEGF, and MMP), as well as immunosuppressive factors (IL-10 TGF-β), that promote the generation of an immunosuppressive cell population and inhibit effector T cells, and also other factors such as chemokines and cytokines that promote metastasis and epithelial–mesenchymal transition (27). Therefore, TAMs represent an important therapeutic target for inhibition at the level of their activation, recruitment, and survival or for the reprogramming of polarization (27, 28). Shibuya et al. showed that multimodal neoadjuvant chemotherapy could decrease the number of immunosuppressive infiltration cells such as myeloid cells (29).

#### Tumor-Associated Neutrophils (TANs)

Analogous to the M1 and M2 dichotomy for TAMs, TANs exhibit a pro-tumor N2 profile with pro-tumor function through the influence of TGF-β (30). Furthermore, pancreatic cancer cells attract neutrophils through the secretion of chemokines, such as CXCL8 and CXCL16 (31). Few studies have evaluated the function of TANs; however, those with the N2 profile have been shown to produce matrix metallopeptidases including MMP-8, MMP-9, neutrophil elastase, reactive oxygen species (ROS), and VEGF and some inflammatory cytokines including TNFα and GM-CSF, which promote tumor and immune cell proliferation (metastatic potential) and favor chronic inflammation (31, 32).

#### Myeloid-Derived Suppressor Cells

Myeloid-derived suppressor cells are a population of cells defined by their immature state, myeloid origin and capacity to suppress the immune response. Through factors in the TME, they can acquire phenotypic and functional characteristics of TAMs and TANs and are thus called mononuclear (Mo-) or granulocytic (G-) MDSCs (14, 31). They are strongly immunosuppressive by their ability to inhibit T-cell proliferation, IFNγ production, and effector T-cell function and to favor Treg generation through the secretion of ROS, Arg1, and iNOS (33, 34). They also promote tumor growth by VEGF and MMP9 secretions. High concentration of MDSCs in the peripheral blood is associated with poor prognosis in PDAC (35).

#### Mast Cells (MCs)

Mast cells can release cytotoxic granules and have the capacity to recruit other immune cell populations by chemokine secretion. They have been found in significantly higher numbers in PDAC compared to those in normal pancreatic tissue (36), where they support tumor growth and angiogenesis and inhibit anti-tumor immunity. The MCs accumulate within the TME, along with macrophages, through the action of tumor-derived chemoattractants such as MCP-1 and RANTE and by tumor-secreted VEGF and FGF (37). MC accumulation correlates with higher tumor grade, diminished survival, and lymph node metastasis.

*In vitro*, MCs induce PDAC cell proliferation and migration (angiogenesis and metastasis) by secreting factors including secretin, VEGF, and IL-8 and tumor growth factors including PDGF and proteases (38, 39).

### Anti-Inflammatory Lymphoid Cells

#### Tregs

In PDAC and solid tumors, CD4^+^ CD25^+^ Foxp3^+^ Tregs are strongly associated with poor prognosis and inversely correlated to the presence of CD8^+^ T cells, with more advanced disease presentation, a lower chance of surgical resection and a poorer survival after resection (36, 40). Patients with PDAC have increased numbers of Tregs. They produce IL-10 and TGF-β and express CTLA-4; thus, they inhibit effector T cells and induce M2 profile TAMs and N2 profile TANs (41).

#### Th17 Lymphocytes

The role of Th17 cells in cancer is highly controversial. Their function seems to depend on the type of cancer, the tumor stage, and the localization (42). In PDAC, while some evidence favors a higher level of Th17 cells in advanced stage tumors, other data in a murine model of pancreatic cancer support Th17 induction increasing survival (43, 44). This inconsistency can be explained by the plasticity of Th17 cells and their ability to promote smoldering inflammation at early stages (45–47). Indeed, Th17 cells are polarized on the one hand by IL-6, IL-23, and Il-1β with pro-inflammatory functions (impact on smoldering inflammation and recruitment of inflammatory immune population) and on the other hand by TGF-β, which induces anti-inflammatory functions (impact on tumor growth, immunosuppressive microenvironment, and angiogenesis) (46, 48). Furthermore, the shift of Th17 to Treg, explained by the plasticity of these cells, is important. In PDAC, patients were shown to exhibit Th17/Treg disorders with higher Treg and lower Th17 cells (49).

#### Th2 Lymphocytes

Th2 cells (GATA-3^+^ IL-13^+^ IL-4^+^), in contrast to Th1 cells, are anti-inflammatory T cells. In PDAC, the TME and CAFs were shown to induce the polarization of Th2 cells by IL-13-mediated dendritic cell secretion *in vitro* (50). The Th2 cells produce IL-13 and IL-4 and thereby induce M2 macrophages or TAMs, which further increases the anti-inflammatory TME. Furthermore, via an amplification loop and T-cell plasticity, Th2 cells inhibit Th1-cell polarization and induce themselves. In tumor tissue, Th2 T-cell infiltrates are a predictive marker of poor prognosis, confirmed by the shift of Th1 to Th2 cells within the TME (13).

#### ɣδ T Cells

ɣδ T cells are “unconventional” T cells. Unlike αβ T cells, these lymphocytes do not require antigen processing and major histocompatibility complex presentation of peptide epitopes. In contrast to current dogma, one study using a mouse model and human samples showed that ɣδ T cells have no anti-cancer properties in pancreatic cancer (51). *In vivo* deletion of ɣδ T cells using a neutralizing antibody resulted in a robust protection against oncogenic progression. The analysis also revealed that infiltrating ɣδ T cells express high levels of T-cell exhaustion ligands (PD-L1 and Galectin-9) and may block the immune response by immune checkpoint inhibition. Altogether these data suggest that, in PDAC, ɣδ T cells promote pancreatic oncogenesis and that their deletion or reactivation could be a novel therapeutic strategy. Surprisingly, the key regulator of Vγ9Vδ2 function BTN3A1 was found to act as a critical marker of PDAC prognosis and is detectable either by IHC or by its soluble receptor sBTN3A1 (52).

### Other Main Anti-Inflammatory Mechanisms

#### Hypoxia

Pancreatic cancer stroma is composed of several main components: CAFs, immune cells and associated cytokines, adipocytes, and endothelial cells. These stromal components are involved in the production of highly toxic conditions including low pH and low oxygen environment (hypoxia). To define the hypoxic status of pancreatic cancer, one study measured tissue oxygenation of the tumor and normal adjacent pancreas during pancreaticoduodenectomy surgery (53). Results of this study showed that PDAC are highly hypoxic compared to normal pancreas.

Cancer cells under hypoxic conditions are more resistant to radiation and chemotherapy (54, 55). This ability to survive is mainly conferred by the hypoxia-inducible pathway involving transcription factors able to induce the expression of several genes controlling cell survival, glycolysis, and other cellular metabolism events. Recent evidence supports the hypothesis of hypoxia being one cause of radioresistance. Indeed, Hajj et al. showed that radiation therapy in combination with TH-302 (a hypoxia-activated pro-drug) allowed tumor growth delay in an orthotopic model of PDAC by comparison with the outcome following these two treatments given separately (56). This TH-302 compound is currently being tested in a pancreatic cancer Phase I clinical trial in combination with Nab-paclitaxel and gemcitabine.

Despite the high levels of hypoxia found in pancreatic cancer, which would be expected to promote angiogenesis, PDAC remains poorly vascularized. This poor vascularization limits blood flow to the tumor and is associated with prominent desmoplasia, which prevents drug delivery and could impede the immune response (57). This hypoxia seems to impact on several escape mechanisms and could therefore be a relevant target for next generation therapeutic options.

#### Pancreatic Stellate Cells (PSCs)

In non-inflamed pancreas, PSCs are resident cells involved in maintaining tissue homeostasis by regulating extracellular matrix turnover (58). During pancreatic injury, quiescent PSCs are activated and transform into myofibroblast-like cells. These activated PSCs secrete extracellular matrix proteins, which generate fibrosis and limit drug delivery to cancer cells (59). Inordinate secretion of extracellular matrix proteins is also linked to hypoxia (see paragraph above) and promotes cancer cell proliferation.

Pancreatic stellate cells can also modulate immune cells via their secretion of cytokines. Indeed, secretion of CXCL12 by activated PSCs reduces the migration of CD8^+^ and CD4^+^ T cells, NK cells, and Tregs to the juxtatumoral compartment within close proximity to the tumor (60). Another study showed that PSCs secreted Galectin-1, which mediated immunosuppression of CD8^+^ T cells and promoted T-cell apoptosis (61). All these data suggest that PSCs could be a good target to enhance immunotherapy for PDAC.

## Immunotherapy in PDAC: State-of-the-Art

Pancreatic ductal adenocarcinoma is currently recognized as one of the deadliest human malignancies. Compared to other cancers, PDAC shows marked resistance to conventional forms of chemotherapy and often develops without early symptoms making its detection and early diagnosis very difficult, greatly limiting treatment capability. No current treatment option has demonstrated long-term benefit in patients with advanced disease who are not eligible for surgery, which represents the majority (80%) of PDAC cases. Although some risk factors have been identified (such as tobacco use, family history of PDAC, and a personal history of pancreatitis, diabetes, or obesity), few patients diagnosed with PDAC have identifiable risk factors (1, 62). For many years, gemcitabine monotherapy was the only treatment available for this cancer (2). More recently, studies found that using gemcitabine in combination with FOLFIRINOX and nab-paclitaxel was more effective than gemcitabine monotherapy (3). Unfortunately, this combination therapy prolonged survival by only a few months and actually increased toxicity.

New therapies are thus urgently needed to combat this highly lethal cancer and further extend the lives of affected patients. Immune-based strategies to treat various cancers during the early stages of development, as well as new immunological approaches to treat advanced disease, are showing significant promise where other approaches have failed (63, 64). In PDAC, potential immunology-based therapies have provided new hope and can be divided into three main subtypes: (i) therapeutic vaccines aimed, as those protecting against infection, to stimulate the immune system to produce tumor-specific T cells and B cells (65); (ii) adoptive therapy in which *ex vivo* expanded cytotoxic cells are injected into the tumor to kill cancer cells (66); and (iii) immune checkpoint inhibitors. After their activation, T cells express “blocker” molecules called immune checkpoints, which allow them to return to normal. Cancer cells divert this blocking mechanism by expressing ligands of immune checkpoint resulting in T-cell anergy. New treatments based on monoclonal therapy have been established to counteract T-cell inhibition by immune checkpoint. Antibodies targeting CTLA4, PD1, and programmed death 1 ligand (PDL1) have demonstrated significant efficacy in non-small-cell lung cancer, renal cancer, and melanoma (67).

Unfortunately, immune checkpoint inhibitor monotherapy targeting these three molecules appears to be ineffective in PDAC (68). One explanation for this resistance could be found in the composition of the immune cell infiltrate. As discussed earlier, several cell subtypes found in the PDAC TME have potent immunosuppressive functions. MDSCs promote pro-tumor macrophages, decrease cytotoxic T cells, and recruit Treg lymphocytes. TAMs inhibit T-cell function and secrete immunosuppressive factors (69). Treg lymphocytes secrete immunosuppressive cytokines (IL-10 and TGF-β) and limit CD8^+^ T-cell activation by the consumption of IL2 available by IL2Rα (70). Together, these cells generate an immunosuppressive environment, which likely interferes with immune checkpoint inhibitors.

Another reason that could explain the immune-based therapy inefficiency is the desmoplastic feature of PDAC stroma caused by hypoxia and TME components, as discussed earlier. Novel therapies targeting these two last obstacles are urgently needed which, when combined with immune checkpoint inhibitors, are expected to provide substantial benefits to patients with PDAC. Furthermore, CTLA4, PD1, and PDL1 may not be the major immune checkpoint molecules involved in immune system inhibition in PDAC. A complete analysis of the immune checkpoint molecules expressed by cancer cells in PDAC could help decipher how immune system inhibition is set up and thus reveal new targets.

Finally, the biology and genetics in PDAC also appear to be very important (11, 71). Indeed, several genetic and transcriptomic studies have demonstrated the classification of PDAC into two or more subtypes including basal versus classic or immunogenic versus non-immunogenic (72). Chen and Mellman recently described cancer-immune phenotyping into the following three different subtypes: the immune-desert, the immune-excluded, and the inflamed tumor (73).

Future immunotherapies should now consider such phenotyping in order to adapt therapeutic strategies to specific groups of patients with the aim of increasing patient survival.

## Conclusion

In PDAC and most solid tumors, the TME and, in particular, the immune network play a pivotal role in their development. From the elimination phase where effector immune cells eliminate and select specific resistant tumor cells to the equilibrium and escape phases, tumor cells induce an immunosuppressive TME. These may be found to target myeloid cells and Tregs, as the most abundant cells in the TME of PDAC. PDAC is a devastating disease that is mostly diagnosed at advanced stages at which strong immunosuppressive immune populations and desmoplastic environment have already developed, likely explaining the inefficiency of current immunotherapies in this cancer. The relation between the PDAC’s biology, genetic, and immune network seems to be very closed and important to adapt therapy for each patient. That is why, further studies are needed to better understand the escape mechanisms relating to immunosuppression in order to reveal the best immune checkpoint therapeutic strategies.

## Authors Note

DO team was labeled “Equipe FRM DEQ 201 40329534.” DO is the senior scholar of the Institut Universitaire de France.

## Author Contributions

EF and CG prepared the manuscript collaboratively with input from SC, JG, JI, and DO.

## Conflict of Interest Statement

DO is the cofounder and shareholder of Imcheck Therapeutics, and JG is the cofounder and shareholder of HalioDx. No potential conflicts of interest were disclosed by the remaining authors. The reviewer YM and handling Editor declared their shared affiliation.
